# The Central Neck Dissection or the Modified Sistrunk Procedure in the Treatment of the Thyroglossal Duct Cysts in Children: Our Experience

**DOI:** 10.1155/2018/8016957

**Published:** 2018-06-19

**Authors:** Beata Pucher, Katarzyna Jonczyk-Potoczna, Agata Kaluzna-Mlynarczyk, Pawel Kurzawa, Jaroslaw Szydlowski

**Affiliations:** ^1^Department of Pediatric Otolaryngology, Poznan University of Medical Sciences, Szpitalna 27/33, 60-572 Poznan, Poland; ^2^Department of Pediatric Radiology, Poznan University of Medical Sciences, Szpitalna 27/33, 60-572 Poznan, Poland; ^3^Department of Pathology, Poznan University of Medical Sciences, Szpitalna 27/33, 60-572 Poznan, Poland

## Abstract

**Background:**

The aim of the study was to present the surgical techniques providing the lowest recurrence rate in treatment of the primary and recurrent thyroglossal duct cyst (TGDC) in children.

**Methods:**

The study included 73 patients operated on because of TGDC in years 2011–2016. Ultrasound was performed in all patients preoperatively. In 8 patients with the recurrence of the disease, the CT or MR was carried out before the surgery. Children with the primary disease underwent the modified Sistrunk procedure. In the revision cases the central neck dissection was a method of choice.

**Results:**

In 45 children, the modified Sistrunk procedure was performed and 28 underwent the central neck dissection. In 2 patients, hematoma occurred after the modified Sistrunk procedure with the need of the surgical revision in one. No complications were observed after the central neck dissection.

**Conclusions:**

A modified Sistrunk procedure is method of choice in the treatment of the uncomplicated TGDC. In selected cases of the TGDC with a history of infected cyst or incision of an abscess or in revision cases the central neck dissection should be considered in order to avoid the risk of the further recurrences.

## 1. Introduction

Congenital neck lesions most commonly found in the pediatric population include the thyroglossal duct cyst and the branchial cleft and arch anomalies. The midline lesions most frequently represented by thyroglossal duct sinus and cyst conditions and dermoid cyst are usually easily distinguished from the more lateral lesions represented by branchial cleft sinus and arch anomalies [1]. Thyroglossal ducts cyst (TGDC)—the most common form of congenital cyst in the neck—consists of epithelial remnants of the thyroglossal tract and presents as a midline neck mass (75% of cases) or slightly off-midline mass (25% of cases). Approximately 80% of TGDC are located at the level or below the hyoid bone and the remaining 20% are situated above the hyoid bone [2]. The mass usually causes no symptoms but may be slightly tender. Often the patient recently suffered from an upper respiratory tract infection which led to enlargement of the mass. During clinical examination the cyst usually moves upward with the tongue protrusion and during swallowing. Males and females are equally affected. The risk of malignant transformation is 1% [3].

The successful management of TGDC is based on an understanding of the embryology and the developmental anatomy of the thyroid gland [4]. Since the wide acceptance of the modified Sistrunk procedure the postoperative recurrence rate has dramatically decreased but still ranges up to 47% [5]. Histological findings have proved that the thyroglossal duct arborizes above and below the hyoid bone so this might lead to such high recurrence rate despite the use of the Sistrunk procedure [6]. The approach proposed by some authors, which is called the central neck dissection, expands Sistrunk's operation to involve the excision of the entire anterior soft tissue compartment. So not only the path of the thyroglossal duct is excised but also possible branching of the duct below and above the hyoid and the lateral course that the duct may sometimes take [4, 7].

## 2. Material and Methods

The study included 73 patients who were operated on because of midline neck mass in the Department of Pediatric Otolaryngology from January 2011 to June 2016. Ultrasonography was the most common diagnostic imaging study which was performed in all patients before the surgery. In 8 cases with the recurrence of the disease the CT or MR was carried out preoperatively (Figure 2).

In children with the primary disease the modified Sistrunk procedure was performed. In cases of infected TGDC, with the fistula or the revision cases of TGDC the central neck dissection was performed.

### 2.1. The Central Neck Dissection Technique

The incision is performed above the cyst. If there is a fistula, the segment of skin is excised with it. The dissection is carefully carried out down to the hyoid bone, but no attempt is made to skeletonize the fistulous tract, so it remains within the central neck dissection tissues. The thick, subcutaneous flaps are elevated superiorly to expose the hyoid bone. The sternohyoid and sternothyroid muscles are transected at the level of the cricoid cartilage and the deep dissection is performed to the pretracheal fascia. Bilaterally, the plane extends to the sternocleidomastoid muscle bordering the width of the anterior compartment. Then, the cricoid and the thyroid cartilages are skeletonized with preservation of the cricothyroid muscle in order to avoid the injury of the cricothyroid space. Above the thyroid notch the fascia covering the preepiglottic space is identified but without entering the space. Then, cephalad to the fascia the inferior border of the hyoid bone is identified. The soft tissue attachments to the bone are removed in a subperiosteal plane with an electrocautery needle and the hyoid bone is skeletonized. The middle third of the hyoid bone is separated from the lateral thirds with the help of bone cutting scissors. The macroscopic examination of the lateral parts of the hyoid bone is always performed—if these parts are thinning or destroyed (sometimes with presence of the tract inside) they are dissected to the level of the healthy bone. The dissection continues posterosuperiorly, with removal of the duct with approximately 10 mm cuff of tissues involving the raphe of the mylohyoid muscles and portion of the genioglossus and hyoglossus muscle immediately adjacent to the foramen cecum. The assistant's gloved finger is introduced intraorally to apply pressure over the base of tongue at the site of foramen cecum to help to guide the dissection. The mucosa of the tongue with a cuff of tissue at the base of tongue is removed without entering the pharynx [3, 4, 7].

## 3. Results

### 3.1. Demographic Data and Clinical Presentation

Forty-three patients were male (58.9%) and thirty were female (41.1%). The median age at the time of admission was 7.2 years.

**Figure 1:**
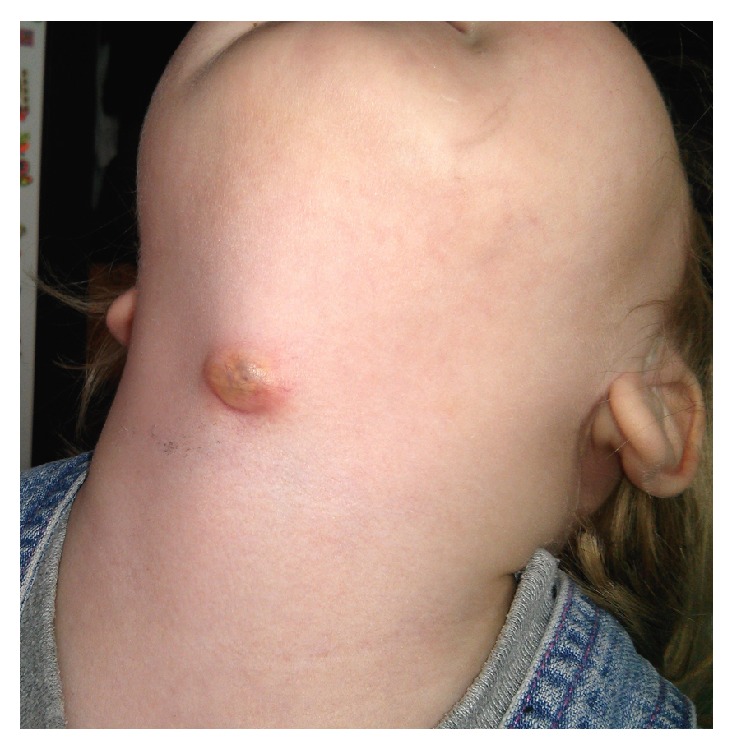
The infected TGDC in 2-year-old girl.

**Figure 2:**
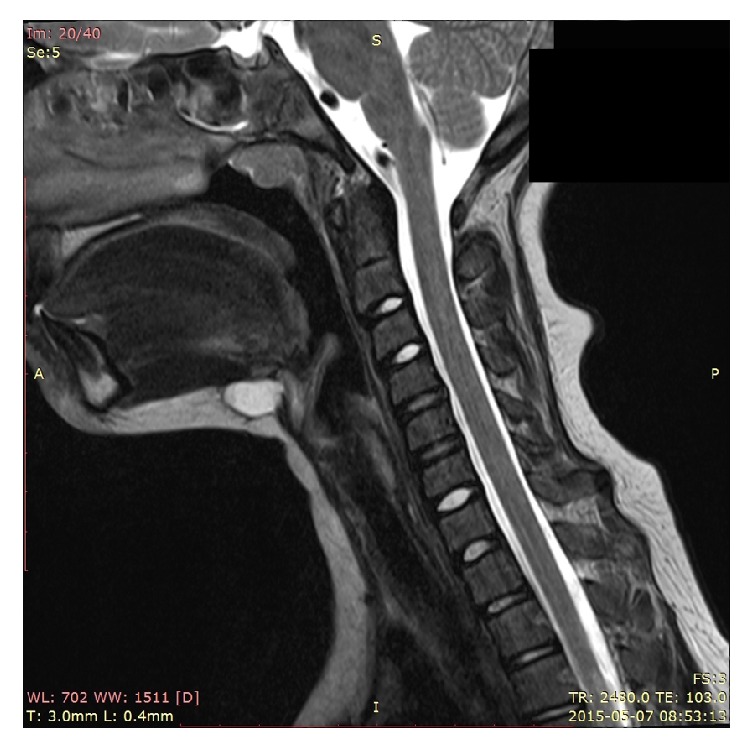
The recurrence of thyrohyoid TGDC in 12-year-old girl in MR imaging.

**Figure 3:**
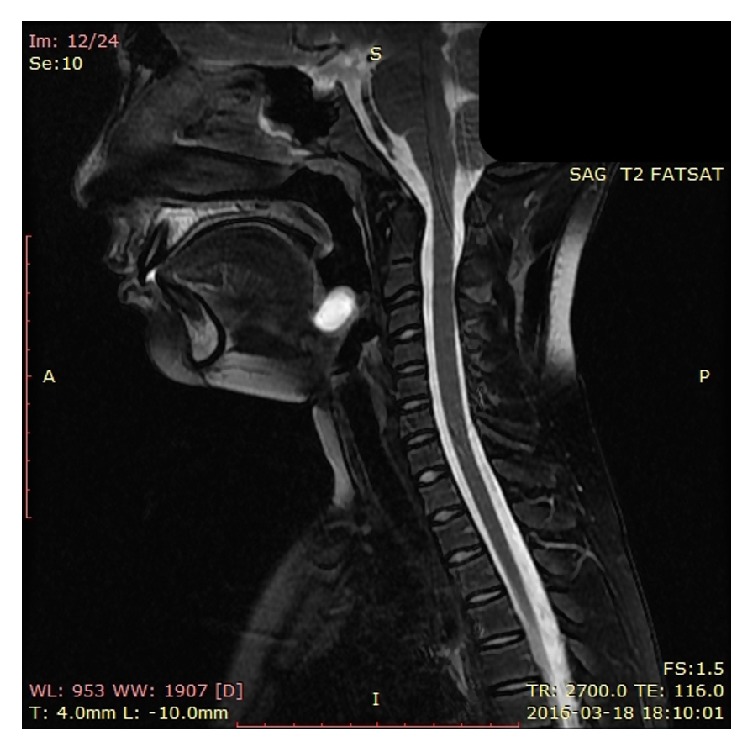
The recurrent lingual TGDC in 11-year-old boy in MR imaging.

In 45 children (61.6%) the main symptom was an anterior neck swelling only and 20 patients (27.4%) presented with a swelling and a draining fistula and history of infection of the lesion (episodes of an abscess incision, recurrent discharge from the fistula) (Figure 1). Eight children (10.9%) required a revision surgery after the simple excision only—6 were operated on in another hospital, and 2 were operated on previously in our department before 2011. The location of the disease to the hyoid bone included the following: thyrohyoid (51 children, 69.9 %) and suprahyoid (21 children, 28.8 %). There was one case with lingual location in our material (1.3%) (Figure 3).

### 3.2. The Surgical Technique

Forty-five children underwent the modified Sistrunk procedure. Children with recurrence of the TGDC and with history of infection of the lesion (28 patients) underwent the central neck dissection surgery (Table 1). In 2 cases the macroscopic evaluation of the hyoid bone revealed the presence of the fistulous tract so 2/3 of the hyoid bone was removed.

### 3.3. The Histopathologic Results

Postoperative diagnosis of the neck mass was based on the result of the histopathologic examination and it confirmed the TGDC in 73 cases.

### 3.4. Complications and Follow-Up

In 2 patients hematoma occurred after the modified Sistrunk procedure. In one case the female patient required surgical revision of the operated region. No complications were observed after the central neck dissection. Every patient was provided with 4 follow-up visits in the outpatient—2 weeks postoperatively, 6–8 weeks after surgery, and 3 and 6 months after treatment. There were no problems with swallowing or phonation observed after the central neck dissection nor the modified Sistrunk procedure. The removal of 2/3 of the hyoid bone did not affect deglutition either. Long-term follow-up till January 2017 revealed good cosmetic effect and no record of the disease recurrence.

## 4. Discussion

Congenital midline neck masses are uncommon lesions with diverse embryogenesis and clinical appearance. Their diagnosis can be very challenging for the pediatric otolaryngologist. The most common of all congenital cystic neck masses is TGDC [6]. In our study it represented 86.9% of all midline neck lesions operated in our department in years 2011-2016. The surgical procedures of all congenital neck lesions represent 1.2% of all surgeries performed in our department annually.

In the diagnostic process, ultrasonography is a very useful modality for thyroid gland and will reveal the cystic nature of a TGDC but does not provide information about the relationship to surrounding structures including hyoid bone. There are three ultrasound features that help to predict and differentiate the TGDC preoperatively: the presence of septae, irregular wall, and solid components within the mass [8, 9]. Although ultrasound is a gold standard in diagnosing TGDC, in all revision cases (8 children) we preferred to perform CT or MR preoperatively which are more accurate in order to avoid further recurrence.

In 1920 Sistrunk suggested that it is necessary rather to excise the core of tissues above the hyoid bone up to the foramen caecum than to separate the canal from the surrounding tissues in order to reduce the recurrence rate [10]. Few years later he modified his method and recommended that the foramen caecum and the tongue mucosa should be left intact and the core of the tissue resected from the suprahyoid region should be of 3 mm diameter [5]. Nevertheless, the recurrence still ranges from 3% to 47% according to various authors [5, 11]. Horisawa et al. explained in 3-dimensional reconstruction the pattern of thyroglossal duct and proved that it tends to branch widely above but also below the hyoid bone which can be a source of recurrence [6, 12]. When the narrow-field approach is used as in the Sistrunk procedure some ductal remnants may not be excised. Also the thorough excision of the tissues of the suprahyoid region is essential. The tract above the hyoid bone is rarely evident on palpation or macroscopic evaluation during the surgery. When the surgeon tries to separate the duct from the surrounding tissues it may also lead to increase of the recurrence rate, especially in patients with previous infections or after the first surgical procedure which was only the simple excision. It is emphasized that after the excision of the hyoid bone the surgeon should watch for the change of the tissue surface from the superior-inferior orientation of the muscles fibers to the smooth and glittering presence which indicates the anterior and lateral margins of resection in the area superior to the hyoid bone [13].

Several authors propose wider neck dissection—called the central neck dissection which involves additionally excision en block of tissues below and above the hyoid bone. There are some concerns about the extent of such operation in pediatric population [4, 7]. But in our material we have also observed that even the excision of the strap muscles and surrounding tissues does not lead to cosmetic defect because of the presence of the thick subcutaneous fat in children which compensates well for the tissue loss.

The major complications after the modified Sistrunk procedure include recurrences, abscess, or hematoma with necessity of surgery, but also entering the airway, the need for tracheotomy, nerve paralysis, hypothyroidism, and death [5]. In our material the hematoma occurred in 2 children after the modified Sistrunk approach and 1 female required an emergency surgical intervention. No complications were observed after the central neck dissection. According to Isaacson's 10 years' experience although the central neck dissection is a safe method, it should not be recommended as a primary procedure because of the risk of the carotid artery and the vagus nerve injury, but also the possibility of the larynx damage [7].

## 5. Conclusions

A modified Sistrunk procedure is method of choice in the treatment of the uncomplicated TGDC. In selected cases of the TGDC with a history of infected cyst or incision of an abscess or in revision cases the central neck dissection should be considered in order to avoid the risk of the further recurrences.

## Figures and Tables

**Table 1:** Surgical technique in patients with neck mass.

Neck mass	No of patients	Surgical procedure	Complications
TGDC	45	The modified Sistrunk procedure	2
TGDC – infected	20	Central neck dissection	0
TGDC recurrence	8	Central neck dissection	0
Total	73	X	2

## Data Availability

The datasets generated during and/or analyzed during the current study are available from the corresponding author on reasonable request.

## References

[1] Tracy T. F., Muratore C. S. (2007). Management of common head and neck masses. *Seminars in Pediatric Surgery*.

[2] Koeller K. K., Alamo L., Adair C. F., Smirniotopoulos J. G. (1999). From the archives of the AFIP. Congenital cystic masses of the neck: radiologic-pathologic correlation. *RadioGraphics*.

[3] Goldsztein H., Khan A., Pereira K. D. (2009). Thyroglossal duct cyst excision-The Sistrunk procedure. *Operative Techniques in Otolaryngology - Head and Neck Surgery*.

[4] Kim M. K., Pawel B. R., Isaacson G. (1999). Central neck dissection for the treatment of recurrent thyroglossal duct cysts in childhood. *Otolaryngology—Head and Neck Surgery*.

[5] Pastore V., Bartoli F. (2014). “Extended” Sistrunk procedure in the treatment of recurrent thyroglossal duct cysts: A 10-year experience. *International Journal of Pediatric Otorhinolaryngology*.

[6] Horisawa M., Niinomi N., Nishimoto K. (1999). Clinical results of the shallow core-out procedure in thyroglossal duct cyst operation. *Journal of Pediatric Surgery*.

[7] Isaacson G. (2001). Central neck dissection for infected or recurrent thyroglossal duct cysts. *Operative Techniques in Otolaryngology-Head and Neck Surgery*.

[8] Oyewumi M., Inarejos E., Greer M.-L. (2015). Ultrasound to differentiate thyroglossal duct cysts and dermoid cysts in children. *The Laryngoscope*.

[9] Inarejos Clemente E., Oyewumi M., Probst E., Ngan B.-Y., Greer M.-L. (2017). Thyroglossal duct cysts in children: Sonographic features every radiologist should know and their histopathological correlation. *Clinical Imaging*.

[10] Sistrunk W. E. (1920). *The surgical treatment of the cysts of the thyroglossal tract*.

[11] Zhu Y.-S., Lee C.-T., Ou C.-Y. (2016). A 16-year experience in treating thyroglossal duct cysts with a “conservative” Sistrunk approach. *European Archives of Oto-Rhino-Laryngology*.

[12] Horisawa M., Sasaki J., Niinomi N., Yamamoto T., Ito T. (1998). Thyroglossal duct remnant penetrating the hyoid bone - A case report. *Journal of Pediatric Surgery*.

[13] Koempel J. A. (2014). Thyroglossal duct remnant surgery: A reliable, reproducible approach to the suprahyoid region. *International Journal of Pediatric Otorhinolaryngology*.

